# Performing wellness, concealing pain: a gendered continuum of challenges for women with lupus in the workplace

**DOI:** 10.3389/fpsyg.2025.1644068

**Published:** 2025-11-12

**Authors:** Armand Bam, Joy Lulema

**Affiliations:** 1 Stellenbosch Business School, Stellenbosch University, Stellenbosch, South Africa; 2 Faculty of Economic and Management Sciences, Stellenbosch Business School, Stellenbosch University, Stellenbosch, South Africa

**Keywords:** systemic lupus erythematosus (SLE), invisible disability, episodic illness, feminist disability theory, embodiment, professional identity, disclosure, workplace inclusion

## Abstract

**Introduction:**

Women with systemic lupus erythematosus (SLE) work under conditions where success is often contingent on concealing pain, managing disclosure, and “performing” wellness in organisational cultures that reward composure over care. Workplaces are not neutral spaces; they are structured by ableist and gendered norms that privilege stability, productivity, and visibility, making episodic illness particularly disruptive.

**Methods:**

This study draws on a narrative inquiry approach with eight professional women living with SLE. Participants were invited to recount their embodied experiences of illness, identity, credibility, and inclusion in the workplace. The narratives were analysed thematically with a feminist disability lens, attentive to the relational and institutional contexts that shape meaning-making.

**Results:**

The findings introduce the Continuum of Embodied Challenges, a conceptual framework tracing the layered tensions participants face in navigating illness and institutional expectations. Diagnosis emerges as both a clinical and epistemic struggle, where uncertainty erodes trust in one’s body. Participants described resisting the category of “disability” even when functionally impaired, and shouldering significant emotional and physical labour to remain credible in spaces that privilege predictability and presence.

**Discussion:**

By centring invisibility, gender, and resistance, this study advances feminist disability perspectives on chronic illness and work. It highlights how episodic disablement is structurally misrecognised in organisations designed around uninterrupted performance. The study argues for institutional models of inclusion that account for bodily unpredictability, fluctuating capacity, and the complexity of living and working with episodic illness.

## 1 Introduction

What does it take to look “well enough” to be believed, and strong enough to stay employed? For women with invisible, episodic conditions like systemic lupus erythematosus (SLE), this question is not rhetorical, it’s routine. Caught between gendered expectations of resilience and the unpredictability of their illness, they navigate professional spaces that reward composure and penalize disruption. Their stories reveal not just the burden of disease, but the labour of appearing undiseased. Yet work itself is not a neutral backdrop to these struggles. Scholars have long shown that workplaces are structured by ableist norms privileging uninterrupted productivity, bodily stability, and linear career trajectories (Jammaers et al., 2016; Sharma et al., 2025). These expectations render illness disruptive not only because of symptoms but because work is organized around assumptions of able-bodiedness (Lundberg and Chen, 2024). For women with SLE, work becomes both a site of aspiration and exclusion, where ambition collides with unpredictability and the performance of wellness is demanded to preserve credibility (Grue, 2024; McGonagle et al., 2024).

In this sense, work is both a site of aspiration and of exclusion. It demands that women with SLE perform wellness, conceal pain, and sustain professional credibility in environments that struggle to accommodate bodily ambiguity (McGonagle et al., 2024). Built on ideals of continuous productivity, visibility, and control, workplaces render illness disruptive not only because of symptoms but because of how professional life is organized around able-bodied norms. Women with SLE thus encounter work as both a site of opportunity and exclusion, where ambition collides with unpredictability, and where the labour of concealing pain becomes integral to sustaining credibility. This paper responds by offering a conceptual framework, the Continuum of Embodied Challenges, that maps the layered tensions women face as they move between diagnosis, identity, and institutional survival.

Systemic lupus erythematosus (SLE) is a chronic, autoimmune disease that affects multiple organs, with symptoms that range from extreme fatigue and joint pain to neurological complications and organ failure (Aringer, 2020). Its episodic and unpredictable presentation defies easy classification. It fluctuates in intensity, eluding conventional diagnostics, and often remains invisible to others. These qualities position SLE within what feminist scholars call “embodied ambiguity,” where the body is both a site of knowledge and a source of social misrecognition. The unpredictability of SLE complicates not only clinical encounters but also the narratives women construct to remain legible and employable in professional spaces.

To explore these complexities, this study draws on narrative inquiry, a method grounded in the belief that people make sense of illness through story (Clandinin and Connelly, 2000; Frank, 1995). Narratives are not merely accounts of what happened; they are how people organise memory, identity, and moral meaning over time. For women with SLE, narrative becomes a way to hold together experiences of disruption, disbelief, and adaptation. It allows insight into how illness is lived in the gaps between flare-ups and how professional identity is reshaped when bodies resist the norms of stability, visibility, and control. Through participants’ stories, this paper traces not only the biographical impact of SLE but the institutional and aesthetic demands that shape how women disclose, endure, and reframe their working lives.

While a growing body of work has examined chronic illness, disability, and the workplace, limited attention has been paid to conditions like SLE that defy categorisation and disrupt normative expectations of visibility, stability, and recovery. Feminist disability scholars have highlighted how disability is shaped not only by bodily limitations but also by social institutions, aesthetic norms, and structural power (Garland-Thomson, 2002; Kafer, 2013). Yet much of this literature continues to privilege visible or physically marked forms of disability. Episodic and invisible conditions remain largely at the margins of these discussions, despite the emotional and logistical complexity they introduce into everyday life.

This gap is particularly acute in studies of gender and professional work. Women are more likely to be diagnosed with autoimmune diseases and more likely to experience dismissal of their symptoms as emotional or exaggerated (Hoffmann and Tarzian, 2001; Quintner, 2020). These demands sit uneasily with the fluctuating and often concealed nature of SLE, reinforcing what (Bertilsdotter Rosqvist et al., 2017) describes as “disabling practices” where work itself amplifies the burden of illness. Existing research on disclosure, stigma, and professional identity has made important strides, but has not sufficiently addressed how individuals navigate the ambiguity of illness in environments structured around ideals of endurance and composure.

This study is situated in South Africa, where a progressive legislative framework exists through the Employment Equity Act (Republic of South Africa, 1998) and the Technical Assistance Guidelines on the Employment of People with Disabilities (Department of Labour, 2004). Despite these frameworks, workplace inclusion for individuals with disabilities remains uneven, often shaped by stigma, organizational cultures, and inadequate accommodations (Bam and Ronnie, 2020). For those with SLE, fragmented healthcare access and the invisibility of symptoms create additional barriers to sustained employment (Bam, 2025b). Existing research has shown how disclosure of chronic illnesses in the workplace is often fraught with tension, with concealment frequently used to protect professional identity (Charmaz, 2010; Gignac et al., 2021; Kulkarni, 2022).

Against this backdrop, examining how women live with, make sense of, and manage SLE in professional life is not only timely but necessary. It opens space to interrogate the gendered, aesthetic, and institutional logics that govern the recognition of illness, and to explore the hidden costs of maintaining credibility, normalcy, and presence in contexts where unwellness is not easily named, seen, or accommodated. Crucially, it also requires problematizing work itself. Rather than treating professional life as a neutral stage on which illness plays out, this study recognizes work as an institution structured by ableist and gendered assumptions about productivity, stability, and presence (Jammaers et al., 2016; Lundberg and Chen, 2024; McGonagle et al., 2024).

Accordingly, and following the problematization of work outlined above, the study is guided by the following research question: How do individuals with systemic lupus erythematosus narrate their experiences of disclosure, identity, and work within the South African context?

## 2 Literature review

The complex realities of living with chronic, invisible illnesses such as systemic lupus erythematosus (SLE) demand interdisciplinary attention. While a range of scholarship explores disability, chronic illness, and gender, there remains limited engagement with how these dimensions intersect in the everyday lives of women navigating professional environments under conditions of fluctuating health, uncertainty, and social misrecognition. In particular, work itself has rarely been problematized as an institution structured by ableist and gendered assumptions that privilege stability, productivity, and presence. This review draws together feminist disability theory, literature on diagnostic delay, tensions between medical authority and lived experience, and work on gendered embodiment to situate the critical terrain from which more nuanced inquiry is emerging.

### 2.1 Feminist disability theory and the politics of visibility

Feminist disability theorists have long argued that disability is not merely a physical impairment but a condition that is produced and shaped by social, institutional, and cultural forces (Garland-Thomson, 2002). Within this tradition, disability is understood as a form of embodied difference that is problematised in relation to normative ideals of independence, productivity, and bodily control. Kafer (2013) expands this framework by highlighting the temporal dimensions of disability, where assumptions about futurity, recovery, and progress inform social responses to illness. These critiques have opened space for thinking beyond the binary of disabled and non-disabled and toward more fluid, relational understandings of impairment.

Yet despite these developments, feminist disability scholarship has largely centred on visible and static forms of impairment (Schalk and Kim, 2020; Thorneycroft, 2024; Wilde and Fish, 2023). Less attention has been paid to episodic and invisible conditions that elude straightforward categorisation, those which shift over time, fluctuate in intensity, and remain medically contested. This relative absence reflects a broader discomfort with bodies that fail to offer clear diagnostic markers or consistent symptom patterns, leaving individuals in a liminal space where they are neither visibly ill nor fully acknowledged as healthy (Beech, 2011). The challenge posed by such conditions underscores the need to examine how social and institutional expectations around disclosure, competence, and appearance shape the experiences of those living with invisible illness (Bam, 2025b).

### 2.2 Invisible illness, delayed recognition, and epistemic injustice

Invisible illnesses are often characterised by subjective symptoms pain, fatigue, cognitive fog that resist objective verification. These features frequently lead to delayed diagnoses, as biomedical systems rely heavily on measurable indicators and reproducible signs (Quintner, 2020; Toro et al., 2020). For conditions such as SLE, whose manifestations vary widely and appear inconsistently, diagnosis is often a process of exclusion, misinterpretation, and emotional distress (Lisnevskaia et al., 2014). Women in particular are disproportionately affected by such delays, due in part to gendered assumptions about the emotional origins of pain and fatigue (Hoffmann and Tarzian, 2001).

The concept of epistemic injustice (Fricker, 2007) is helpful in understanding these experiences. When patients report symptoms that are difficult to substantiate medically, their knowledge is frequently devalued, leading to both clinical inaction and internalised doubt (Werner and Malterud, 2003). In the context of invisible illness, this dynamic compounds the uncertainty already produced by the body’s unpredictability (Dobson, 2021). Delayed recognition not only disrupts access to care, it destabilises identity, erodes confidence in an individual’s perceptions, and complicates participation in social and professional life. While existing research has offered valuable insights into the medical pathways of diagnostic delay (Beusterien et al., 2013; Faugno et al., 2024), it has paid less attention to how delayed recognition affects individuals’ ability to navigate roles that demand composure, dependability, and clarity, particularly in professional environments that reward consistency and penalise ambiguity.

### 2.3 Medical authority, patient knowledge, and narrative fracture

The divide between medical expertise and lived experience is well-established in the sociology of health and illness (Bury, 1982). Chronic illness often challenges the dominant biomedical model, which privileges objectivity, linearity, and curability (Babik and Gardner, 2021; Charmaz, 2002). Instead, those with ongoing, complex conditions must construct narrative coherence in the face of fragmentation and disbelief. Frank (1995) identifies this as the “quest narrative” a way of making sense of suffering by turning illness into a site of meaning rather than merely a biomedical disruption.

However, this process is rarely straightforward. Chronic, invisible illnesses produce what Frank (1995) also calls “narrative wreckage,” where individuals struggle to reconcile their embodied knowledge with the limited frameworks offered by medical professionals. The tensions between clinical dismissal and lived legitimacy frequently shape not just how individuals experience illness, but how they communicate, seek help, and construct public and professional selves (Bam, 2025a; Fava and Petri, 2019). The literature has not fully examined how this narrative tension unfolds in the workplace, where employees are expected to appear reliable, healthy, and emotionally contained, often without openly acknowledging illness or instability.

### 2.4 Gender, appearance, and the burden of bodily control

The management of bodily appearance is a central concern in gendered expectations of professional competence. Feminist scholars have noted that women’s bodies are sites of both scrutiny and discipline, particularly in contexts where leadership or competence is linked to composure and self-control (Bordo, 2003; Wolf, 1991). Illness, especially when it manifests in visible ways like rashes, swelling, or hair loss disrupts these carefully managed presentations.

Goffman’s (1963) concept of stigma and the practice of “passing” remain relevant in contemporary work environments where individuals with non-apparent conditions must decide how much of themselves to reveal. Invisible illnesses complicate this decision-making process, as they can often be hidden, while choosing to conceal them demands significant emotional and physical labour (Berkley et al., 2019). The literature has demonstrated that women with chronic illness frequently engage in “appearance management” (Werner et al., 2004), aiming to minimise disruption to workplace norms. However, these studies rarely explore how such efforts affect long-term identity development, access to leadership, or sustainability of work.

Moreover, research on chronic illness and gender has predominantly emerged from Western contexts and has not sufficiently addressed the structural and cultural dynamics that shape embodiment and self-presentation in African and other Global South settings. Issues of race, class, and cultural expression intersect with illness in ways that remain insufficiently theorised in mainstream academic literature. These gaps also draw attention to the need to problematize “work” itself. Work is not a neutral arena but an institution structured by ableist and gendered assumptions that privilege stability, productivity, and normative bodily appearance (Bertilsdotter Rosqvist et al., 2017; Jammaers et al., 2016). For women with episodic and invisible conditions such as SLE, these institutional logics amplify exclusionary pressures, framing fluctuation as disruption rather than as part of the ordinary variability of human embodiment.

### 2.5 Theoretical framing

The study is guided by identity theory in two complementary strands (Jenkins, 2008) relational social identity (identity negotiated through social interaction) and Stryker and Burke’s (2000) identity control/ verification model (identity meanings maintained through role salience and feedback) and is enriched by feminist disability studies (Garland-Thomson, 2002; Kafer, 2013; Thorneycroft, 2024; Wilde and Fish, 2023). Identity theory highlights how individuals construct and negotiate self-understanding through social interaction (Stryker and Burke, 2000). This lens is particularly useful for examining how disclosure or concealment of chronic illness shapes professional identity (Lejzerowicz, 2017). Feminist disability scholarship extends this perspective by interrogating how embodied difference is situated within relations of power (Garland-Thomson, 2002). It emphasises how structural and cultural ableism renders illness identities invisible or devalued, shaping how individuals internalise stigma or resist erasure (Bam, 2025b).

Analytic map. In the discussion, diagnostic delay is read as breakdown of identity verification (Stryker and Burke, 2000), the management of bodily appearance as ongoing interactional identity work (Jenkins, 2008), disability ambivalence as strategic identity management under power (Kafer, 2013), and workplace navigation as an institutional test of identity stability (Garland-Thomson, 2002).

Taken together, these perspectives position disclosure not as a single decision but as an ongoing negotiation of identity within workplaces that often privilege able-bodied norms. This framing enables the study to show how narratives of living with SLE reflect broader struggles of visibility, legitimacy, and belonging.

## 3 Methodology

### 3.1 Design and approach

This study employed a qualitative, narrative inquiry approach to explore the professional lives of women diagnosed with systemic lupus erythematosus (SLE). Narrative inquiry, as articulated by Clandinin and Connelly (2000), centres lived experience as stories and situates personal narratives within broader social, cultural, and institutional contexts. This approach was particularly suited to understanding how women make sense of chronic illness through their work lives, how they navigate disclosure, and how they negotiate the tension between ambition and health. Rather than examining discrete “cases,” the study attended to individual narrative trajectories, each shaped by time, relationality, and context (Chase, 2005).

The aim was not to generalise across a population, but to surface the meanings participants assign to their experiences, capturing both shared patterns and the situated uniqueness of each woman’s story. This interpretive orientation aligns with narrative researcher’s concern for temporality, voice, and the ethics of representation (Riessman, 2005).

### 3.2 Participant recruitment

Participants were recruited using a snowball sampling method (Etikan, 2017). Initially participants were identified through the researchers’ professional and academic networks. These individuals referred others in similar roles or industries who met the inclusion criteria: a formal SLE diagnosis, experience of paid employment, and a willingness to share personal narratives. Given the private and sometimes stigmatized nature of illness disclosure, this approach was effective for accessing a hard to reach population and building relational trust necessary for narrative inquiry.

### 3.3 Participants as narrative contributors

In line with narrative inquiry’s emphasis on storied lives and contextualised meaning-making (Clandinin and Connelly, 2000), the brief participant profiles are included here not as findings, but as part of the methodological framing. These vignettes offer insight into the participants’ professional backgrounds, illness trajectories, and self-representations elements that shape how they narrate experience. Including these profiles supports what (Riessman, 2005) calls “narrative positioning,” helping others to understand the social, relational, and occupational contexts in which meaning is constructed. They also provide a forward orientation to the themes developed in the findings section, where participants’ accounts are analysed more deeply.

#### 3.3.1 Participant 1: the principal who wouldn’t let go

A former school principal in her late 50s, Participant 1 was diagnosed with SLE in the 1990s, long before public awareness of the disease had grown. She often worked through extreme pain and fatigue, determined not to be seen as less capable than her peers. Even as her body began to resist her schedule, she continued showing up “rolling out of bed” on difficult days, refusing to ask for relief. She eventually disclosed her condition to her headmaster, who responded with compassion and flexibility, allowing her to skip physically demanding duties. Still, the internalised pressure to match others’ performance never left her. Her story reflects both the cost of silent endurance and the possibility of care when leadership responds with trust.

#### 3.3.2 Participant 2: the corporate strategist with lupus fog

An operations manager in her 40s, Participant 2 indicated she was a “sharp thinker” and known for her high-functioning leadership. Her SLE diagnosis came after a long period of medical misdirection, including treatment for arthritis. While she described herself as “blessed with good managers,” she admitted that disclosure required her to educate them about lupus, something she had to learn about herself while navigating the mental fog and exhaustion the disease brought on. Despite support, she remained cautious about who she told. “I do not want people to feel sorry for me,” she said, balancing the desire for empathy with the need to retain authority in a demanding environment.

#### 3.3.3 Participant 3: the teacher who wrote her truth

Diagnosed in her teens, Participant 3’s experience of SLE shaped her identity early. She spoke of hair loss, rashes, and deep fatigue during adolescence symptoms that confused even doctors. Now a high school teacher, she used her writing to make sense of her journey. Publishing a memoir about her illness meant her colleagues already knew about her condition. While this offered relief from disclosure anxiety, it also introduced the risk of being defined by her diagnosis. Her professional path is one of visibility, resilience, and tension between self-expression and stigma.

#### 3.3.4 Participant 4: the quiet achiever in finance

A chartered accountant with over 15 years in one role, Participant 4 carefully concealed her diagnosis for most of her career. “I just go on,” she said. “If my knees hurt, I do not complain I’m just quiet.” A single mother to an autistic child, her professional decisions were shaped as much by caregiving as by her own health. Despite being headhunted multiple times, she chose to stay in a job that felt “safe,” resisting ambition in favour of emotional and physical sustainability. Only after proving her work ethic did she feel safe enough to disclose. Her vignette reveals the tension between over-performance, maternal duty, and the fear of being disqualified for needing care.

#### 3.3.5 Participant 5: from heels to hospital

An accountant in her 30s, Participant 5’s diagnosis arrived without warning. Her case of lupus affected her peripheral nervous system, a rare and disabling manifestation. Despite this, she returned to work after 9 months and was met with overwhelming support. Her colleagues fetched her from the train station daily; her employer ensured she continued to be paid. Her story defies the assumptions that illness always isolates, it showed how collective care and inclusive practice can make recovery a shared effort.

#### 3.3.6 Participant 6: the buyer who kept the balance

Working in procurement, Participant 6 struggled with chronic fatigue, headaches, and hives symptoms initially dismissed as allergies. She appreciated having managers who took her health seriously but wrestled with the feeling of being “a special case.” The advent of hybrid work post-COVID helped her manage flare-ups discreetly, yet she worried about perceptions from coworkers unaware of her condition. She declined a promotion, fearing additional stress. Her story illustrates the emotional labour of invisibility, the relief of flexibility, and the quiet fear of being misunderstood.

#### 3.3.7 Participant 7: the HR professional who put herself last

A human resources manager, Participant 7 only recently began to reckon with how much she had deprioritised her own well-being. Reflecting on her choices, she said, “I’m actually last on the list.” She had disclosed her diagnosis to her manager, who responded with kindness and openness. Still, she often pushed herself to show up, even when unwell. Her narrative is a turning point, an attempt to unlearn guilt, seek balance, and recognise that self-compassion is a form of leadership too.

#### 3.3.8 Participant 8: the storyteller in admin

A bid administrator known for her “outgoing nature,” Participant 8 wore her illness lightly but lived its weight deeply. She designed a t-shirt campaign to raise lupus awareness and used her encounters with strangers as moments of education. Yet in the workplace, she often pushed through severe symptoms swollen joints, fatigue, and skin flare-ups without letting others know. “You want more but you do not know what that more is,” she said, voicing the tension between potential and protection. Her story reflects advocacy, ambiguity, and the yearning to be seen beyond what the body conceals.

### 3.4 Data collection

Data were collected through in-depth semi-structured interviews that allowed participants to shape the direction and flow of their narratives while exploring guiding topics such as career history, flare-up management, disclosure decisions, support systems, and professional identity (Clifton et al., 2018; DiCicco-Bloom and Crabtree, 2006). Interviews ranged from 60–120 min conducted online or in private in person settings. All interviews were recorded with consent, and transcribed verbatim to ensure accuracy and depth. Participants’ names were anonymised using pseudonyms.

In line with the narrative approach, interviews were treated as co-constructed conversations rather than one-directional question and answer sessions (Riessman, 2005). Reflexive attention was given to the role of the researchers in shaping how stories were told and participants were encouraged to use their own words, metaphors, and pacing. This approach not only generated depth but also laid the foundation for the analytic focus on voice, tone, and narrative flow described in the data analysis.

### 3.5 Data analysis

Although thematic analysis (Braun and Clarke, 2006), provided the guiding framework for identifying patterns, the analytic process remained grounded in the narrative structure of participant accounts. Narratives were treated as co-constructed texts with attention paid not only to the content but also to voice, tone, metaphor, and the emotional weight of key events (Riessman, 2005). Researcher 1 transcribed the interviews and conducted initial line-by-line coding to capture emotionally and temporally meaningful units (e.g., “pushing through fatigue,” “concealing symptoms,” “watching my body change”). These were shared and refined with Researcher 2. Codes were grouped into thematic threads that represented common narrative tensions: identity, visibility, work performance, and bodily disruption. Theme names were drawn from participants’ own words or metaphors to preserve voice. Reflexivity was maintained throughout via a research journal (Watt, 2015), and no software was used to ensure immersion in the material. The analysis was recursive, with constant movement between coded units, narrative flow, and context (Riessman, 2005).

While narrative inquiry provided the overarching frame, thematic analysis (Braun and Clarke, 2006) was integrated to identify cross-patterns. This dual approach enabled us to retain the richness of individual stories while generating broader insights across cases. We recognise that thematic coding risks fragmenting narrative coherence. To mitigate this, extended participant quotes were preserved, member checking was conducted, and counter-stories that did not fit dominant themes were retained to illustrate diversity. For example, Participant 5’s account of sudden collapse into ICU, followed by a rare experience of collective workplace care, did not align neatly with the broader themes of concealment and disruption. We retained this as a counter-story to highlight the heterogeneity of experiences and to avoid presenting illness trajectories as uniform.

Alternative strategies, such as developing narrative typologies (e.g., “success stories” or “struggle stories”), were considered. Such an approach would have highlighted the coherence of individual cases. Treating each participant’s story as a distinct type. While valuable, we ultimately selected thematic analysis to better capture the cross-cutting tensions of work and disclosure. This decision aligned with our theoretical framing of identity as relational and fluid (Burke and Stets, 2009; Jenkins, 2008), and allowed us to trace patterns of concealment, negotiation, and resistance without reducing participants’ accounts to fixed categories.

### 3.6 Ethics statement

Anonymity was ensured using pseudonyms, and participants could review or withdraw their data at any stage. Ethical clearance was granted by the Stellenbosch University Rec: Social Behavioural and Education Research (No: 31352), and informed consent was obtained prior to data collection. The study adhered to the guidelines of the Protection of Personal Information Act (POPIA) and offered optional psychological support in case of emotional distress.

## 4 Findings

This study examined how women living with systemic lupus erythematosus (SLE) experience, interpret, and navigate illness within professional spaces. The findings foreground the emotional, embodied, and institutional complexity of living with an invisible and episodic condition in a world structured around visibility, predictability, and composure. The themes of confusion and delayed recognition, bodily disruption, discomfort with disability, and workplace navigation emerged from the storied accounts and reveal the multidimensional labour women undertake to maintain both identity and credibility. Each theme is rooted in participants’ own framing of their lives and was constructed to reflect the emotional, temporal, and relational complexities of living with SLE.

### 4.1 Confusion and medical recognition

The theme captures the confusion, fear, and gradual loss of physical control experienced by participants as they navigated unexplained symptoms in the early stages of the disease. The invisible nature of symptoms, the emotional strain accompanying not knowing, and the initial disconnect between their physical experience and medical recognition contributed to a delayed recognition and often misdiagnosis of the disease.

For many participants, the onset of symptoms came well into adulthood, after they had established their careers. This made the lack of answers especially unsettling as they experienced managing a body in decline and navigating a healthcare system that seemed equally unsure. The absence of familiarity with lupus among healthcare providers deepened the confusion. Participant 1’s (Teacher) account reflects the widespread sense of uncertainty surrounding lupus as “it was something like gambling around you.” Instead of clarity, “doctors were not used to this kind of sickness… giving you this and that.’ Similarly, Participant 2, (Operations Manager), was treated for arthritis until her doctor suspected “something else.” Her onward referral experience “was stressful” as she was shared the “concerning news” being diagnosed with lupus while never having heard of Lupus before. For Participant 3, (Teacher), her GP also felt that “something is just not right” and then received a diagnosis from a specialist, whose “only advice was do not eat spicy food.”

Uniquely, Participant 3’s (Teacher) story contrasts with others who encountered lupus in adulthood, showing how the disease can quietly embed itself early, altering a life’s trajectory long before diagnosis. Her experience reveals the slow creep of the disease and offered insight into what it means to grow up with a body that sends distress signals that no one can interpret. Her symptoms began quietly with “little things, once in a while, that could not be explained.” like fatigue, weight loss, dry skin without context or clarity.

What makes her narrative distinct is how illness seeped into adolescence, a period already marked by change and uncertainty. For her, lupus did not arrive with urgency it arrived subtly, blending into the background of growing up. But even without a name, it disrupted her physical stability and began to shape how she related to her body. The phrase “rearing its ugly head” captures how unpredictable and invasive the condition felt.

Significantly, Participant 5 revealed how SLE can erase the line between wellness and crisis in a matter of hours. For her, she moved from the poised confidence of a normal workday, dressed, capable, mobile to lying immobile in an ICU bed. The dramatic shift captures the deep uncertainty SLE imposes, from minor discomfort to life-threatening episodes.

“I’m wearing my 12-inch stilettos and then after a while I can feel something is not right. Next thing, when I wake up, I am in ICU. I could not move from the neck all the way down to my toes.” (Participant 5, Accountant).

The extreme contrast and hidden intensity of the illness highlighted how participants had to contend with managing daily tasks and the sudden withdrawal of their bodies cooperation with no warning. This diagnostic uncertainty not only delayed care but fractured participants’ trust in their bodily experience raising tensions of epistemic injustice and medical credibility.

### 4.2 Disruption of bodily identity

Participants described the significant confusion of losing control over their bodies in ways that defied logic or expectation resulting in a disorienting experience of body certainty. This loss was not only functional but existential, disrupting their sense of normalcy, identity, and trust in their own bodies. This disconnection from previously effortless abilities created fear and disbelief, especially in the absence of a diagnosis. The sudden shift where strength turned to fragility overnight left them feeling as if they were watching their own decline without explanation.

“I could not understand. I was continuously falling ill, tired. I wanted to get out of bed, and I could not, I said to my husband I literally cannot move. Picking up a piece of paper was difficult, it feels too heavy, people do not understand that.” Participant 2, (Operations Manager).

Lupus did not just disable, it altered self-perception, relationships, and the rhythms of everyday professional life. Hair loss, facial rashes, swelling, and weight changes affected how participants saw themselves and how others responded to them. For the women, these changes were deeply entwined with identity, confidence, and their femininity. These visible disruptions, though medically explained, were socially loaded. Participants carried not only the physical effects of lupus, but the burden of not meeting gendered expectations of beauty, health, and professional presentation.

“One day I went to the hairdresser… she said do you know you have got big bald patches on your head.” (Participant 4, Chartered Accountant).

“I would wake up, my eye would be this big. I’d lose my hair. I had this terrible rash on my fingers.” (Participant 3, Teacher).

The invisibility of symptoms led to disbelief and minimisation from others and sometimes from themselves. Participants described a jarring disconnection between how they appeared and what they felt. This created tension between maintaining a polished, feminine exterior and managing the chaos beneath the surface. The pressure to remain composed, attractive, and productive even while in pain reveals how femininity becomes an additional site of labour under chronic illness.

“I just started swelling from my ankles… I thought maybe it’s water retention.” (Participant 8, Bid Administrator).

The effort to conceal symptoms or rationalise them through socially acceptable explanations (stress, allergies, overwork) was described as indicative of gendered expectations to cope quietly and remain composed. Participant 6, Senior Buyer experienced “headaches for a year” thinking she “must not inconvenience work” while “everyone would say it’s allergies acting up…” For many, their work environments required them to push through symptoms in order to appear capable and competent. This was especially hard when flare-ups impacted physical presentation or energy levels.

‘I do not know where I got the energy. I never even had someone to fill in for me, except other teachers who took subjects in my class.’ Participant 1, (Teacher).

‘It was very challenging because I was sick quite a lot, but I still made it to work, I still showed up and stuff, but again, stretching experience, challenging growth.’ Participant 3, (Teacher).

Moreover, the mental struggles of Participant 7, (Human Resources) was evident and at times admittedly destructive as she pushed herself to show up at work:

“I walked into the office and people are like, oh my gosh, what are you doing, because I was still wobbly, you know? I said to them, I’m here to get well and to work. I need to get my mind off these things.”

Navigating these work landscapes just like the journey leading up to diagnosis, came with physically, mentally and emotionally interrelated factors that participants had to navigate. This theme reveals how chronic illness unsettles not only bodily function but gendered and professional identity the key tensions of embodiment, stigma, and workplace performance discussed later.

### 4.3 Discomfort with disability

For women living with lupus, the term “disability” did not easily align with their lived experiences or how they wanted to be perceived by others or by themselves. While most participants accepted lupus as an invisible illness, they pushed back against being defined by a word that, to them, signified limitation, dependency, or social exclusion. For participants language itself became a site of tension between illness and identity, between recognition and resistance.

Participants understood that lupus was a condition others could not see but they also believed that invisibility should not equate to incapability. While they acknowledged the daily impact of symptoms like fatigue, pain, and swelling, they were reluctant to claim or be assigned the identity of “disabled.”

“No, I never [heard of the term invisible disability]… but I have this illness, but it does not make me any less than anybody.” (Participant 1, Teacher).

There was a strong sense of self-worth and resistance to being diminished by a label. Many saw “disability” as socially constructed around visible impairment, dependency, or being unable to meet expectations. In contrast, they viewed themselves as fully capable, even if managing an internal battle. Participant 3 reinforced this perspective, using the more neutral term “invisible disease.” For her, the struggle wasn’t in accepting illness it was in constantly having to explain its hidden nature.

“A lot of lupus patients do not look sick… people will say, but you look fine.” (Participant 3, Teacher).

This disconnection between appearance and reality contributed to a deep ambivalence: while they experienced real physical limits, they also feared being misread as unfit, unwell, or unreliable if they identified as disabled. The word did not match the image they worked hard to project. For a few participants, their view of disability shifted from being a personal identity to a legal and institutional category that could be used to access support. Through professional roles or self-driven learning, they came to see disability as something that, when disclosed, could unlock workplace rights.

“I only found this out because we were trying to do a disability drive… If I am going to disclose my disability, whatever it is I am dealing with I want something in return.” (Participant 6, Senior Buyer).

The participants framing was found to be pragmatic where once identified, it became a strategic tool. Some women were willing to engage with the term “disability” not out of comfort, but out of necessity using it to negotiate access to accommodations like time off, flexibility, or medical support. Participant 2, Operations Manager, suggested she had *“looked it up”* and that you *“can register this as a disability”* revealing the need to balance her medical reality with social perception as people said she did *“not behave like someone that has lupus.”*

In the end, these women navigated two worlds one where lupus qualified as a disability under labour policies, and another where their daily performance hid any trace of it. Their stories reveal that disclosure was never a simple personal choice, it was shaped by organisational culture, leadership response, and the invisible boundaries of inclusion. Despite institutional constraints, participants enacted subtle forms of resistance, delaying promotions, prioritising wellness, or redefining ambition, which points to moments of agency and recalibration. These layered dynamics of concealment, negotiation, and strategic self- presentation form part of a broader cycle of tensions, taken up in the discussion and visually mapped in the final figure.

## 5 Discussion

The narratives presented in this study offer more than accounts of illness; they expose the layered contradictions women must navigate when professional expectations collide with invisible, episodic disruption. While each participant’s experience of systemic lupus erythematosus (SLE) was shaped by unique circumstances, their stories revealed shared tensions: how to appear capable while managing pain, how to disclose without inviting doubt, and how to maintain ambition when the body defies planning. These complexities sit at the intersection of gender, chronic illness, and work, where the unspoken demands of reliability and control meet the unpredictable rhythms of fatigue, stigma, and structural rigidity. The discussion that follows extends each of the three findings themes through a feminist disability lens, offering both theoretical grounding and a conceptual synthesis.

### 5.1 Diagnostic delay as epistemic and embodied rupture

The experiences of confusion, misdiagnosis, and disbelief that participants recounted reflect longstanding critiques of diagnostic delay in invisible illness (Werner and Malterud, 2003). However, beyond the clinical implications, this delay functioned as a disruption of bodily certainty and narrative coherence. For many, the journey to diagnosis was marked by doubt internally absorbed and externally reinforced, which echoes Fricker’s (2007) concept of epistemic injustice. Participants’ embodied knowledge was dismissed until legitimised by clinical confirmation, underscoring the tension between lived experience and biomedical authority (Bury, 1982; Frank, 1995). In identity-theoretic terms, this constitutes a failure of identity verification (Stryker and Burke, 2000), without congruent feedback, participants could not stabalise a “well” identity, pushing them into fragile, negotiated space (Jenkins, 2008) associates with ongoing social identity work.

These narratives support feminist critiques that the credibility of women’s symptoms continues to be undermined by gendered assumptions within medical discourse (Hoffmann and Tarzian, 2001). When participants described being told their symptoms were minor, hormonal, or stress-related, it became clear that the delay in recognition was not just about the disease’s complexity, it was about whose knowledge counted. While diagnostic accuracy is important, this study shows that the emotional aftermath of being disbelieved shapes how women subsequently relate to healthcare systems, their own bodies, and their professional environments.

### 5.2 The body as both private and public site of disruption

The disruption of bodily identity was more than a physiological shift; it was an existential and aesthetic one. Participants described losing trust in their body’s reliability, but also in its appearance, swelling, hair loss, and facial rash symptoms that undermined not only function but femininity. These experiences affirm that gendered embodiment plays a central role in how chronic illness is lived and managed (Charmaz, 1995; Werner et al., 2004). The professional space, in particular, became a site where bodies were constantly monitored for signs of weakness or instability.

Drawing on Goffman’s (1963) concept of stigma, the women’s accounts highlight how invisible illness imposes a demand to pass as well. Yet unlike Goffman’s framework, this passing was not simply about concealing difference; it was about performing normalcy while internally negotiating breakdown. This labour is both emotional and material, requiring participants to push through flare-ups, avoid disclosure, and maintain outward composure. The strain of this dual performance, appearing capable while managing dysfunction, illustrates the gendered expectations of resilience in professional spaces (Bordo, 2003; Wolf, 1991). Here, identity work is both interactional and performative: women actively sustain competence displays in everyday encounters (Jenkins, 2008) while managing role expectations whose confirmation is required to keep identity meanings intact (Stryker and Burke, 2000).

### 5.3 Disability as an unclaimed or strategic identity

While feminist disability theory has advanced critical understandings of impairment as socially constructed (Garland-Thomson, 2002; Kafer, 2013), it has often focused on visible, stable forms of disability. This study supports emerging critiques that feminist disability scholarship has not adequately accounted for episodic and invisible conditions (Bailey and Mobley, 2019; David, 2025; Wilde and Fish, 2023). This ambivalence reflects strategic identity management: concealment and selective disclosure operate as tactics to preserve role credibility (Stryker and Burke, 2000) within interactional contexts where the “disabled” identity category carries status risk (Jenkins, 2008). Consistent with Kafer (2013), disability functions less as a fixed self-descriptor than as a contingent, power-mediated resource that can be resisted or mobilised.

Most participants rejected the label of “disabled,” associating it with incapacity, loss of agency, and being viewed as less competent. For them, disability was not an identity, but a misreading, one that threatened their professional legitimacy. Some, however, began to view disability status as a pragmatic resource, a means to access workplace accommodations or wellness protections. This dual relationship with disability, as both resisted and strategically claimed, reveals a tension that is not captured in binary models of disabled/non-disabled identity. It underscores the need for a feminist disability framework that embraces fluctuation, contradiction, and contingency (Kafer, 2013). Participants’ discomfort with the label did not stem from shame alone, it reflected a deeper critique of how disability is constructed in the workplace as a risk, rather than a reality.

### 5.4 Institutions of work and the conditions of inclusion

Across the narratives, it became clear that disclosure was not only a personal decision it was shaped by organisational culture, leadership responsiveness, and structural flexibility. While some participants described empathetic line managers who adapted schedules or duties without hesitation, others experienced subtle discouragement or were forced to justify existing accommodations. These findings align with research showing that inclusion in professional spaces is often discretionary, uneven, and dependent more on interpersonal goodwill than institutional design (Dahanayake et al., 2018; Faruk, 2024).

The participants’ reluctance to disclose illness also supports the work of Gignac et al. (2021), who show that people with episodic conditions often fear being perceived as unreliable. In professions where presence, punctuality, and performance are tightly policed, disclosure becomes a risk, a potential trigger for pity, lowered expectations, or exclusion. This reinforces the argument that work itself must be problematised, rather than functioning as a neutral space, it is organised through ableist and gendered logics that reward stability and penalise fluctuation (Jammaers et al., 2016; Lundberg and Chen, 2024). Within such a framework, SLE is not only a medical challenge but a structural contradiction, as women must constantly negotiate legitimacy in systems designed without them in mind. Organisational responses thus become the feedback environment for identity verification (Stryker and Burke, 2000): supportive leadership stabilises role identities; discretionary or sceptical climates destabilise them, intensifying the ongoing interactional labour (Jenkins, 2008) describes.

### 5.5 Reclaiming agency and reframing capacity

Despite these challenges, participants showed considerable agency. They redefined success in ways that accommodated their health without surrendering ambition. Some chose stability over promotion; others delayed career changes to prioritise recovery. These choices reflect a broader critique of the “ideal worker” model, rooted in ableist and masculinist assumptions of linear progress and unbroken availability (Acker, 1995; Goffman, 1959; Norstedt, 2019). These recalibrations illustrate identity agency: by adjusting role salience and redefining acceptable feedback, participants actively re-author the meanings attached to “competent worker,” aligned with Stryker & Burke’s view of identities as managed control systems within interactional settings. Rather than interpreting these women’s decisions as resignation, they should be read as resistance to institutional norms that fail to recognise non-normative embodiments of leadership and labour.

It is from these acts of resistance and negotiation alongside the tensions mapped in participants’ narratives that the Continuum of Embodied Challenges emerged as a conceptual framework. Developed from a synthesis of the narrative and conceptual threads in this study, the framework captures how women with SLE navigate professional settings while negotiating fluctuating bodies, contested identities, and institutional expectations.

### 5.6 Continuum of embodied challenges for women with SLE in the workplace

**Figure 1:**
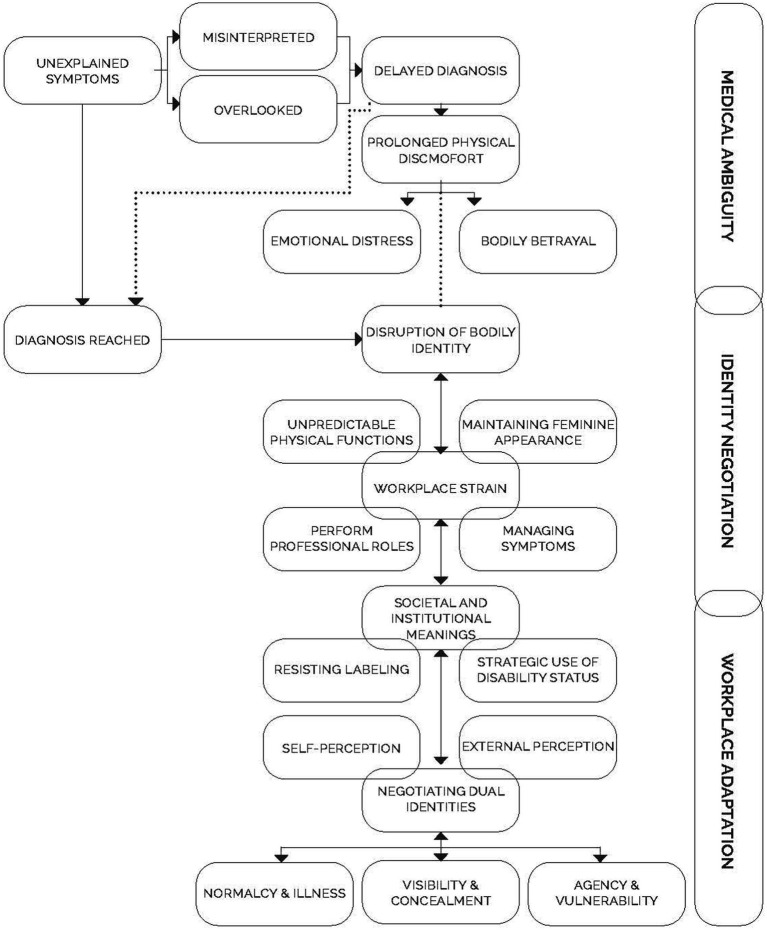
Continuum of embodied challenges for women with SLE in the workplace. The figure maps three overlapping and recursive zones, medical ambiguity, identity negotiation, and workplace adaptation, highlighting how women cycle between bodily disruption, self perception, concealment/visibility, and professional role performance across time.

The Continuum of Embodied Challenges is presented here as a tentative empirically grounded framework developed from participant narratives. While it maps recurring tensions across diagnosis, identity, and workplace navigation, it should be understood as provisional, requiring further empirical testing and refinement. Rather than a linear sequence, participants moved back and forth along the continuum, at times revisiting diagnostic doubt, bodily disruption, or disclosure struggles across career stages, indicative of the fluid and recursive nature of these challenges. This fluidity reflects the episodic and unpredictable nature of lupus and the ways identity negotiations are continually reworked over time. Moreover, the Continuum of Embodied Challenges illustrates a dynamic, multi-directional flow of experiences, where women engage in active meaning-making, resist reductive labels, and negotiate legitimacy within both healthcare and workplace systems (See Figure 1).

Figure 1 visually synthesises these tensions into three interconnected zones, medical ambiguity, identity negotiation, and workplace adaptation, each populated by overlapping experiences. Within medical ambiguity, participants recounted unexplained symptoms, delayed diagnosis, prolonged discomfort, and emotional distress. Moving into identity negotiation, the disruption of bodily identity, the management of appearance, resistance to labels, and the balancing of normalcy/ illness highlight the recursive labour of sustaining credibility. Finally, workplace adaptation captures how participants managed symptoms, performed professional roles, and strategically deployed or resisted disability status while navigating vulnerability and agency. The arrows underscore that these are not sequential stages but recursive dynamics; a flare up or managerial change could pull participants back into earlier zones mirroring the episodic and unpredictable course of lupus.

## 6 Conclusion

This study offers new insight into how women with systemic lupus erythematosus (SLE) negotiate the intersection of illness, identity, and work. While existing feminist disability theory has interrogated the social construction of impairment and the institutional regulation of bodies, much of this scholarship has focused on visible, stable, or mobility-related disabilities. In contrast, this study foregrounds the experience of a fluctuating, invisible condition one that often escapes recognition in both medical and organisational spaces. Drawing on identity theory, relational identity as interactional negotiation (Jenkins, 2008) and identity verification through role-based feedback (Stryker and Burke, 2000) concealment emerges not simply as avoidance but as a strategic act of identity management, allowing participants to preserve credibility in professional settings. Feminist disability studies further illuminate how these acts are embedded in structures of power that normalise able-bodiedness, rendering chronic illness identities invisible of devalued (Kafer, 2013, 2019). These insights extend prior research on workplace disclosure (Bam and Ronnie, 2020; Kulkarni, 2022) by showing how identity work unfolds in the specific context of South Africa, where formal legislative protections coexist with exclusionary workplace cultures.

The findings reveal how women with SLE constantly balance credibility with concealment, ambition with fatigue, and composure with inner collapse. Their experiences illustrate the compounded challenges of navigating a condition that is medically misrecognised, socially minimised, and professionally unsupported. Taken together, these dynamics underscore the need to problematize work itself, professional life is not merely the stage on which illness is negotiated but a central site of ableist and gendered norms that marginalise bodies marked by fluctuation and ambiguity (Grue, 2024; Jammaers et al., 2016). This reframing contributes to feminist disability studies and critical organisational scholarship by positioning work as a contested institution that must adapt, rather than expecting individuals alone to accommodate.

What emerges is not a linear narrative of illness nor a closed cycle of recurrence, but rather a Continuum of Embodied Challenges that captures the recursive tensions women face across diagnosis, disruption, disclosure, and professional engagement. This framework demonstrates how women redefine ambition not by relinquishing it, but by negotiating its terms amidst fluctuating bodily realities and institutional constraints. In theoretical terms, the continuum operationalises identity theory by showing how verification and negotiation processes are destabilised by episodic illness (Stryker and Burke, 2000; Jenkins, 2008), while also extending feminist disability scholarship to account for invisible and contingent embodiments (Kafer, 2013; Garland-Thomson, 2002). Participants resisted the disabling gaze while strategically engaging with disability frameworks to secure necessary accommodations. Their narratives challenge narrow conceptions of the “ideal worker” and invite broader understandings of capacity, consistency, and professionalism.

Moreover, the framework maps how diagnostic ambiguity, identity disruption, institutional culture, and strategic disclosure are interrelated, fluid processes. This framework offers researchers, practitioners, and policymakers a tool for understanding the dynamic, relational nature of chronic illness in professional contexts. It emphasizes the importance of designing workplaces that recognise the labour of invisibility and embrace inclusion as a continuous, flexible practice.

Future research could expand on these insights by further testing and theoretical refinement to clarify mechanisms and scope, as well as exploring variation across race, class, gender identity, and organisational cultures. Longitudinal studies and comparative approaches would further illuminate how individuals and institutions co-construct responses to episodic illness. Ultimately, this study calls for a reframing of professionalism itself, one that makes space for vulnerability, adaptation, and the full humanity of those who work while unwell, and that requires organisations to adapt their design and expectations to fluctuating embodiments rather than placing the burden solely on individuals.

## Data Availability

The datasets presented in this article are not publicly available due to the sensitive and personal nature of the narrative data collected. Requests for access to anonymized data may be considered on a case-by-case basis and must be submitted to the relevant institutional ethics committee, in accordance with approved ethical protocols and participant confidentiality agreements. Requests to access the datasets should be directed to AdenWilliams, aden@sun.ac.za.
